# Non-canonical PI3K-Cdc42-Pak-Mek-Erk Signaling Promotes Immune-Complex-Induced Apoptosis in Human Neutrophils

**DOI:** 10.1016/j.celrep.2016.09.006

**Published:** 2016-10-04

**Authors:** Julia Y. Chu, Ian Dransfield, Adriano G. Rossi, Sonja Vermeren

**Affiliations:** 1 The MRC Centre for Inflammation Research, Queen’s Medical Research Institute, 47 Little France Crescent, University of Edinburgh, Edinburgh EH16 4TJ, UK

## Abstract

Neutrophils are peripheral blood leukocytes that represent the first line of immune cell defense against bacterial and fungal infections but are also crucial players in the generation of the inflammatory response. Many neutrophil cell surface receptors regulate important cellular processes via activation of agonist-activated PI3Ks. We show here that activation of human neutrophils with insoluble immune complexes drives a previously uncharacterized, PI3K-dependent, non-canonical, pro-apoptotic signaling pathway, FcγR-PI3Kβ/δ-Cdc42-Pak-Mek-Erk. This is a rare demonstration of Ras/Raf-independent activation of Erk and of PI3K-mediated activation of Cdc42. In addition, comparative analysis of immune-complex- and fMLF-induced signaling uncovers key differences in pathways used by human and murine neutrophils. The non-canonical pathway we identify in this study may be important for the resolution of inflammation in chronic inflammatory diseases that rely on immune-complex-driven neutrophil activation.

## Highlights

• Immune-complex-activated human neutrophils use PI3Kβ/δ-Cdc42-Pak-Mek-Erk signaling
• Immune-complex-induced non-canonical neutrophil signaling is pro-apoptotic
• Other immune-complex-induced neutrophil functions depend on alternative PI3K effectors
• Immune-complex-induced PI3K signaling is not conserved between humans and mice

## Graphical Abstract

**Figure:**
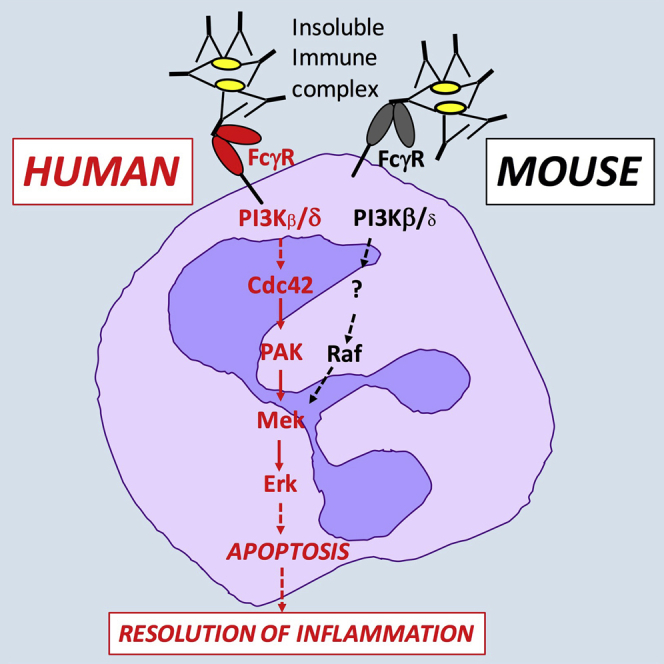

## Teaser

Dysregulated PI3K signaling is associated with many disease processes. Chu et al. now find that an unconventional signaling pathway, PI3Kβ/δ-Cdc42-Pak-Mek-Erk, regulates immune-complex-induced apoptosis in human neutrophils. This non-canonical PI3K signaling pathway, which is not conserved in the mouse, may affect the resolution of inflammation in humans.

## Introduction

Neutrophils, the most abundant circulating leukocytes in humans, represent the first line of immune cell defense against bacterial and fungal infections. Neutrophils are also a key component of the inflammatory response (Nathan, 2006, Nauseef and Borregaard, 2014). Activated neutrophils leave the bloodstream to migrate to sites of infection or sterile insult. However, uncontrolled neutrophil activation can contribute to significant host tissue damage, as evidenced in a number of chronic inflammatory diseases such as rheumatoid arthritis and proliferative glomerulonephritis. Neutrophils are terminally differentiated, short-lived cells that are programmed to undergo apoptosis. Plasma membrane alterations associated with neutrophil apoptosis trigger phagocytic clearance by macrophages. This prevents the release of pro-inflammatory cell debris as a consequence of secondary necrosis, limiting host damage, and is crucial for the resolution of inflammation (Michlewska et al., 2007, Poon et al., 2014).

Neutrophils are activated by a variety of extracellular stimuli, including formylated bacterial peptides and immune complexes, that bind specific cell surface receptors. This induces intracellular signaling cascades that initiate tightly controlled effector functions. Immune complexes are important mediators of neutrophil recruitment and neutrophil-dependent tissue damage in many inflammatory diseases, including rheumatoid arthritis, systemic lupus erythematosus, and proliferative glomerulonephritis (Mayadas et al., 2009). Immune complexes activate neutrophils and induce a range of effector functions, including the formation of reactive oxygen species (ROS), degranulation and cytokine production, as well as neutrophil apoptosis (Fossati et al., 2002b, Gamberale et al., 1998, Ottonello et al., 2001, Schettini et al., 2002). Neutrophils bind soluble and insoluble as well as immobilized immune complexes via their immunoglobulin G (IgG) Fc receptors (FcγRs). FcγR ligation induces intracellular signaling, with receptor proximal events including activation of Src/Syk kinases and several key downstream signaling pathways, including protein kinase C, phospholipase Cγ, and agonist-activated phosphoinositide 3-kinases (PI3Ks) (van Rees et al., 2016).

Agonist-activated PI3Ks are key regulators of cellular signaling that are involved downstream of many cell surface receptors, including FcγRs. Because dysregulated PI3K signaling is associated with many diseases, including neutrophil-dependent chronic inflammatory conditions, PI3K signaling is the focus of both basic research and drug discovery programs. Four isoforms are known, PI3Kα, β, γ, and δ, all of which are expressed by the neutrophil. Following activation, agonist-activated PI3Ks produce the lipid second messenger phosphatidylinositol (3,4,5)-trisphosphate (PIP3) by phosphorylating the plasma membrane component phosphatidylinositol (4,5)-bisphosphate. In the neutrophil as elsewhere, PI3Ks signal through multiple downstream effectors to regulate numerous aspects of neutrophil biology (Hawkins et al., 2010). Despite this, the analysis of PI3K signaling has often focused on the best-characterized PI3K effector, Akt (also known as protein kinase B [PKB]), and indeed, Akt phosphorylation is often used as a readout of PI3K activity.

The present project set out to characterize signaling processes downstream of agonist-activated PI3Ks in the neutrophil. Using a combination of pharmacological inhibition, activity assays, and functional assays, we identified a non-canonical pathway, PI3K-Cdc42-Pak-Mek-Erk that operates in immune-complex-stimulated human neutrophils. This pathway is pro-apoptotic, regulating the ratio of the Bcl-2 family members Mcl-1 and Bax. The present work furthermore uncovered significant differences between signaling pathways employed by human and mouse neutrophils.

## Results

### PI3K Lies Upstream of Erk in Immune-Complex-Stimulated Human and Mouse Neutrophils

**Figure 1:**
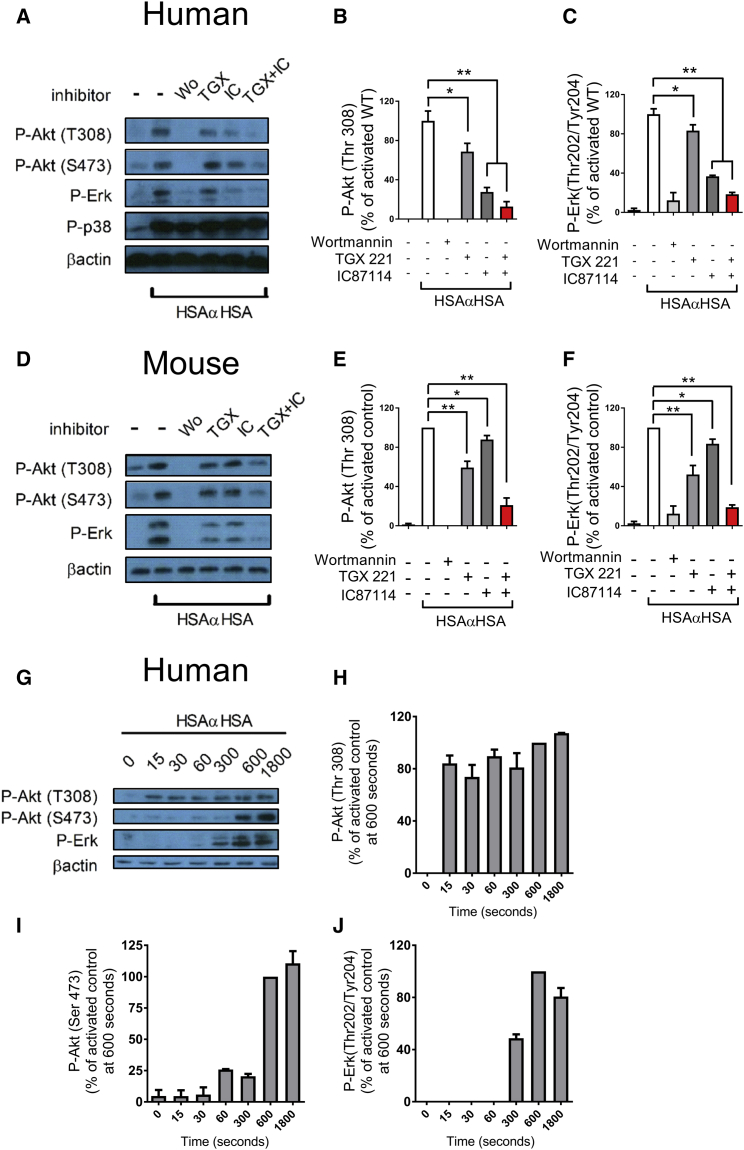
Erk Is Activated Downstream of PI3K in iIC-Stimulated Neutrophils (A–J) Peripheral blood-derived healthy donor neutrophils (A–C and G–J) or bone marrow-derived mouse neutrophils (D–F) were pre-incubated with PI3K inhibitors (wortmannin, pan-PI3K; TGX221, PI3Kβ-selective; IC87114, PI3Kδ-selective) or vehicle at 37°C for 10 min as indicated prior to stimulation with 10 μg/mL iIC (HSAαHSA) or buffer for 10 min (A–F) or the indicated time points (G–J). To terminate the assay, cells were pelleted, followed by resuspension in ice-cold lysis buffer. Soluble protein was subjected to SDS-PAGE and western blotting to detect specific phosphorylation events or β-actin as a loading control as indicated. Representative examples are shown together with densitometry data integrated from a minimum of three separately conducted experiments. For ease of viewing, the data shown are normalized to the activated control. Error bars show SEM. ^∗^p < 0.05, ^∗∗^p < 0.01. See also Figure S1.

We stimulated human and mouse neutrophils with insoluble immune complexes (iICs) and observed significant PI3K (as determined by Akt phosphorylation) as well as Erk and p38 mitogen-activated protein kinase (MAPK) activation. Interestingly, Erk but not p38 MAPK activation was completely PI3K-dependent in both mouse and human neutrophils, as indicated by the use of the pan-PI3K inhibitors wortmannin (Figures 1A–1F) or LY294002 (data not shown). PI3K-dependent Erk activation was also observed with neutrophils that had been stimulated by being plated onto integrin ligands or onto immobilized immune complexes (Figure S1). Comparison of the roles of PI3Kβ and δ in immune-complex-activated human neutrophils revealed that, in contrast to mouse neutrophils (Kulkarni et al., 2011), PI3Kδ rather than PI3Kβ made the major contribution (Figures 1A–1F).

Akt is known to be regulated by PI3K in two ways: directly, via PDK1-dependent phosphorylation of Thr 308 (Alessi et al., 1997, Stephens et al., 1998), and also indirectly, via mTORC2-dependent phosphorylation of Ser 473 (Sarbassov et al., 2005). We next examined the timing of Erk phosphorylation with that of both phosphorylation sites on Akt. Thr 308 phosphorylation of Akt occurred within 15 s of stimulation of neutrophils with iICs, whereas Ser 473 phosphorylation was detectable only after 10 min, peaking 30 min after stimulation. In contrast, phosphorylation of Erk1/2 on Thr 202 and Tyr 204 became apparent at 5 min and peaked 10 min after iIC stimulation (Figures 1G–1J). Thus, PI3K-dependent Erk stimulation was an indirect event that was regulated independently of PI3K-mediated phosphorylation of Akt.

### PI3K-Dependent Erk Activation of Human Neutrophils Involves Mek but Not Ras or Raf

**Figure 2:**
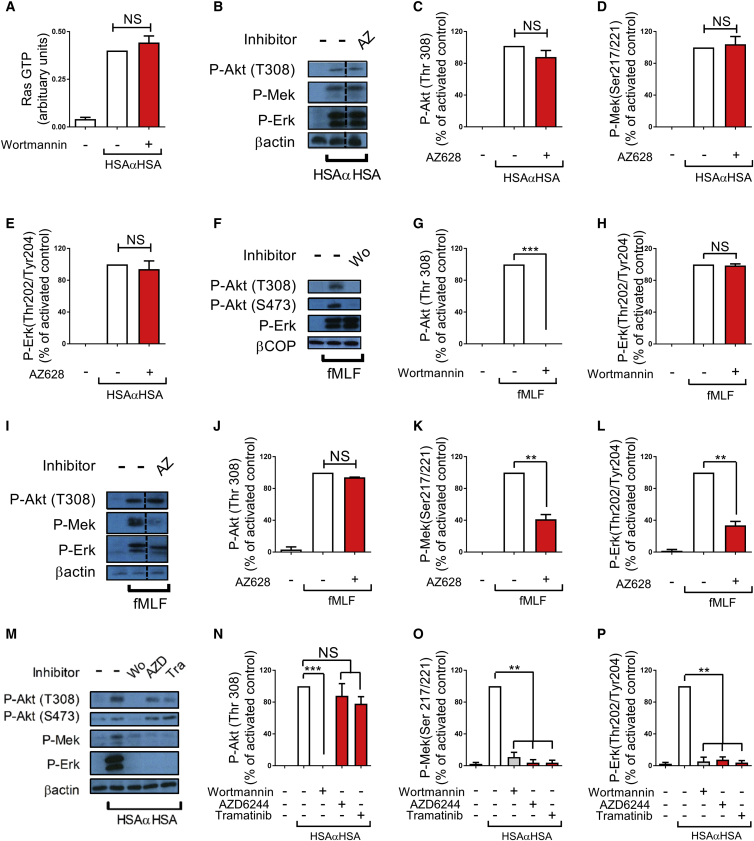
Erk Lies Downstream of Mek but Not Raf or Ras in iIC-Stimulated Human Neutrophils (A–P) Peripheral blood derived healthy donor neutrophils were pre-incubated with small-molecule inhibitors (AZ628, Raf-specific; AZD6244 and tramatinib, Mek-specific) or vehicle at 37°C for 10 min as indicated prior to 10-min stimulation with 10 μg/mL iIC (HSAαHSA) (A, B–E, and M–P) or 60-s stimulation with 100 nM fMLF (F–L). To terminate the assay, cells were pelleted, followed by resuspension in ice-cold lysis buffer. Soluble cellular protein was subjected to (A) analysis of guanosine triphosphate (GTP)-Ras by G-LISA assay or (B–P) SDS-PAGE and western blotting to detect specific phosphorylation events or β-actin or β-COP as a loading control as indicated. Representative blots are shown (B, F, I, and M) together with densitometry data integrated from a minimum of three separately conducted experiments. For ease of viewing, the data shown are normalized to the activated control. Error bars show SEM. The dotted lines in (B) and (I) indicate where lanes derived from the same blot were pasted next to one another for ease of viewing. NS, not significant. ^∗∗^p < 0.01, ^∗∗∗^p < 0.001.

Our observation of PI3K-dependent Erk activation was unexpected because activation of Erk usually follows the canonical Ras-Raf-Mek-Erk cascade. We therefore sought to further characterize this pathway. Ras is known to activate PI3Kα, δ, and γ but not β (Burke and Williams, 2015), although, under certain conditions, PI3K has been demonstrated to lie upstream of Ras (Wennström and Downward, 1999). Because no specific Ras inhibitors have been reported yet, we examined Ras activity in iIC-stimulated neutrophils in the presence or absence of wortmannin to test whether PI3K might lie upstream of Ras in iIC-stimulated neutrophils. iICs drove substantial activation of Ras, which was not affected by inhibition of PI3K (Figure 2A), in line with the notion that Ras is activated independently or indeed upstream of PI3K following FcγR stimulation.

We next tested the involvement of Raf upstream of Erk by making use of a covalent Raf inhibitor, AZ628 (Hatzivassiliou et al., 2010). In these assays, Raf inhibition had no effect on Akt and Erk (or Mek) phosphorylation of iIC-stimulated neutrophils (Figures 2B–2E). Because these results were unexpected, we undertook a comparative analysis of fMet-Leu-Phe (fMLF)-stimulated human neutrophils, where Erk activation was independent of PI3K (Figures 2F–2H). In contrast to the effects of Raf inhibition on iIC-mediated signaling, Erk and Mek (but not Akt) activation in fMLF-stimulated human neutrophils was attenuated (Figures 2I–2L).

To test the involvement of Mek upstream of Erk, we pre-treated neutrophils with two Mek inhibitors: AZD6244 and trametinib/GSK1120212 (Abe et al., 2011, Yeh et al., 2007), before stimulating them with iICs. Mek inhibition with either of these compounds abolished Mek and Erk but not Akt activation (Figures 2M–2P), in line with the notion that Erk phosphorylation was Mek-dependent. We concluded that PI3K-dependent activation of Erk in iIC-stimulated human neutrophils followed a non-canonical pathway that involves Mek but not Raf or Ras.

### PI3K Signaling Pathways Are Poorly Conserved between Human and Mouse Neutrophils

**Figure 3:**
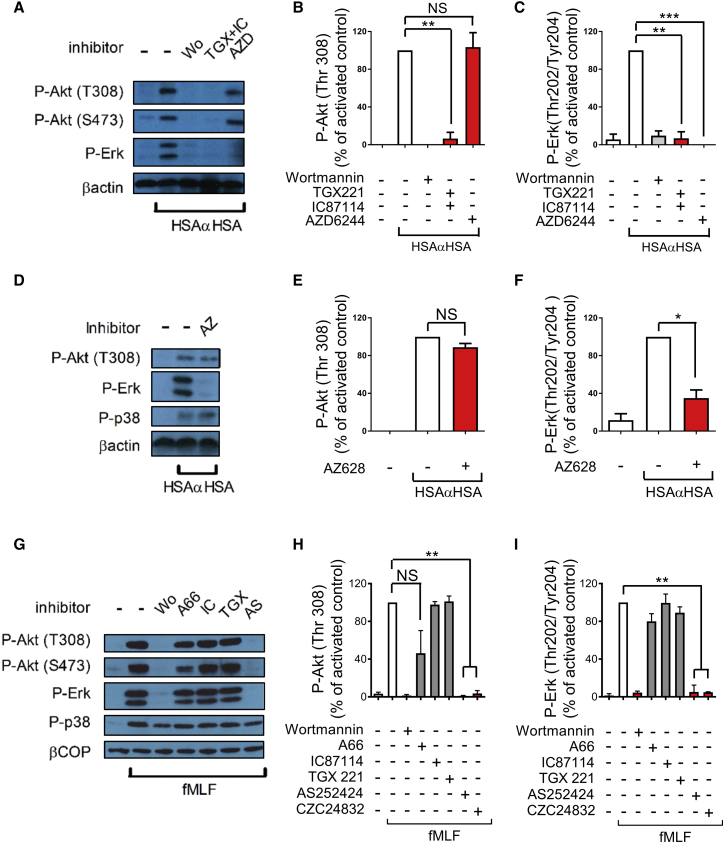
PI3K Signaling Is Not Conserved in Human and Mouse Neutrophils (A–I) Bone marrow-derived mouse neutrophils were pre-incubated with small-molecule inhibitors (Wortmannin, pan-PI3K; TGX221, PI3Kβ; IC87114, PI3Kδ; AZD6244, Mek; A66, PI3Kα; AS252424 and CZC24832, PI3Kγ; AZ628, Raf) or vehicle at 37°C for 10 min as indicated prior to stimulation with 10 μg/mL iIC (HSAαHSA) (A–F), 1 μM fMLF (G–I), or vehicle. To terminate the assay, cells were pelleted, followed by resuspension in ice-cold lysis buffer. Soluble cellular protein was subjected to SDS-PAGE and western blotting to detect specific phosphorylation events or β-actin or β-COP as loading controls as indicated. Representative examples are shown together with densitometry data integrated from a minimum of three separately conducted experiments. For ease of viewing, the data shown are normalized to the activated control. (H) and (I) include an additional PI3Kγ inhibitor, CZC24832, which had not been included in the example shown in (G). Error bars show SEM. ^∗^p < 0.05, ^∗∗^p < 0.01, ^∗∗∗^p < 0.001.

We next examined which signaling pathways were operating in iIC-stimulated mouse neutrophils. We found that, as for human neutrophils, Erk but not Akt phosphorylation was abolished by inhibiting Mek (Figures 3A–3C). However, inhibition of Raf in mouse neutrophils caused partial Erk inhibition (Figures 3D–3F), suggesting that, although PI3K lies upstream of Erk in iIC-stimulated human and mouse neutrophils, different pathways operate in the two organisms.

In support of our suggestion that there is differential signaling in human and mouse neutrophils, analysis of fMLF-stimulated mouse neutrophils revealed that Erk but not p38 MAPK activation was completely PI3K-dependent, further contrasting the situation in human neutrophils. Use of isoform-specific inhibitors, including the PI3Kγ-selective AS252424 (Pomel et al., 2006) and CZC24832 (Bergamini et al., 2012), suggested that this was due to PI3Kγ (Figures 3G–3I). Moreover, although inhibiting Mek abolished Erk (but not Akt) activation (data not shown), inhibiting Raf in fMLF-stimulated mouse neutrophils reproducibly caused partial Erk inhibition (data not shown).

### PI3K Regulates Erk via Non-canonical Pak Signaling

**Figure 4:**
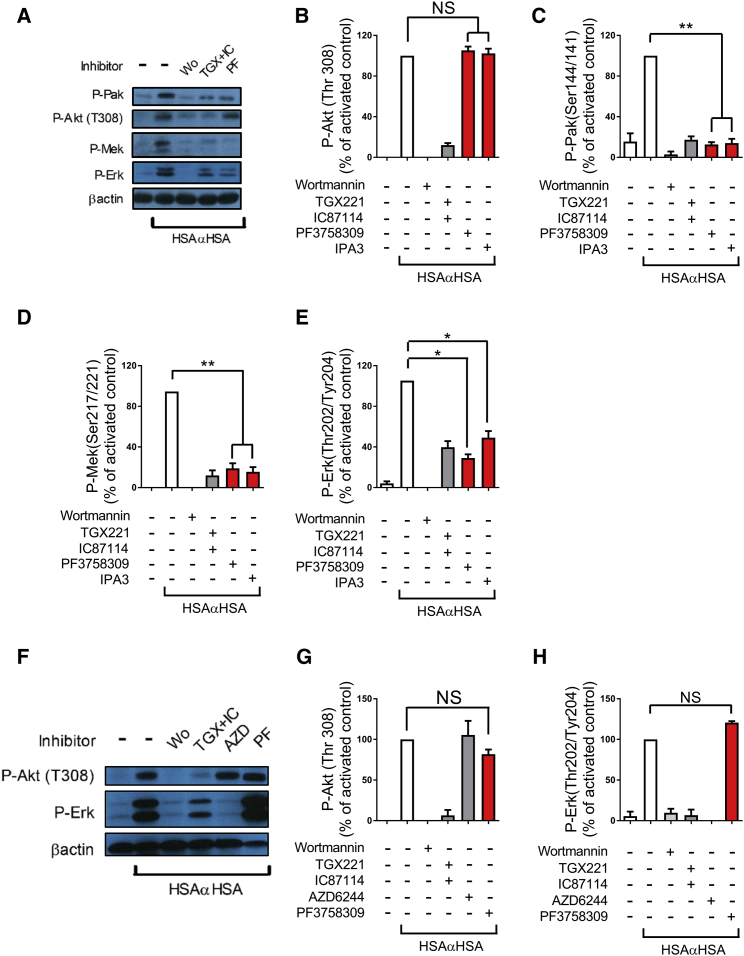
Human but Not Mouse Neutrophils Use Pak as Mek Kinase following iIC Stimulation (A–H) Peripheral blood-derived healthy donor neutrophils (A–E) and bone marrow-derived mouse neutrophils (F–H) were pre-incubated with inhibitors (PF3758309 and IPA3, Pak inhibitors) or vehicle at 37°C for 10 min as indicated prior to stimulation with 10 μg/mL iICs (HSAαHSA) or vehicle. To terminate the assay, cells were pelleted, followed by resuspension in ice-cold lysis buffer. Soluble cellular protein was subjected to SDS-PAGE and western blotting to detect specific phosphorylation events or β-actin as a loading control as indicated. Representative examples are shown together with densitometry data integrated from a minimum of three separately conducted experiments. For ease of viewing, the data shown are normalized to the activated control, and error bars show SEM. Note that the additional Pak inhibitor IPA3 shown in (B)–(E) was not included in the example shown in (A). ^∗^p < 0.05, ^∗∗^p < 0.01.

We next sought to define the involvement of alternative Mek kinases. p21-activated kinase (Pak) has been demonstrated to function as a Mek kinase in a number of contexts, including in myeloid cells (Eblen et al., 2002, Smith et al., 2008). In our experiments with human neutrophils, Pak was phosphorylated in a PI3K-dependent fashion on Ser 144 following stimulation with iICs (Figures 4A and 4C), in line with a stimulatory Pak autophosphorylation event (Chong et al., 2001). We further tested Pak’s potential involvement by using a pan-Pak inhibitor, PF3758309 (Zhao and Manser, 2010), and a Pak1-3 inhibitor, IPA3 (Deacon et al., 2008). Both compounds significantly inhibited Mek and Erk (but not Akt) activation in human neutrophils that had been stimulated with iICs (Figures 4A–4E).

We tested the Pak inhibitors and Pak phosphorylation with mouse neutrophils that had been stimulated with iICs. Pak inhibition did not interfere with Akt or indeed Erk activation in iIC-stimulated mouse neutrophils (Figures 4F–4H) or indeed those stimulated with fMLF (data not shown). We did not observe any Pak autophosphorylation on stimulation of mouse neutrophils with iICs (data not shown). These results suggested an involvement of Pak as MAP kinase kinase kinase (MAP3K) in iIC-activated neutrophils downstream of PI3K in human but not mouse neutrophils. Taken together, our data suggest that signaling pathways downstream of PI3K are poorly conserved between mouse and human neutrophils. In view of these differences between signaling pathways engaged in mouse and human neutrophils, we decided to concentrate on iIC-induced signaling in human cells for the remainder of this study.

### Cdc42 but Not Rac Is Activated in a PI3K-Dependent Fashion in iIC-Stimulated Human Neutrophils

**Figure 5:**
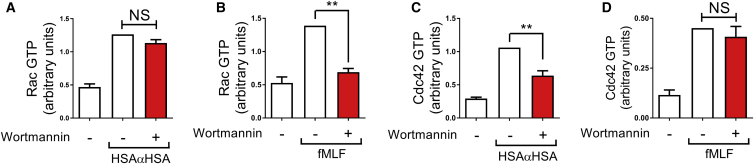
Cdc42 but Not Rac Is Activated in a PI3K-Dependent Fashion in iIC-Stimulated Human Neutrophils (A–D) Peripheral blood-derived healthy donor neutrophils were pre-incubated with the pan-PI3K inhibitor wortmannin or vehicle at 37°C for 10 min as indicated prior to stimulation with 10 μg/mL iICs (A and C), 100 nM fMLF (B and D), or buffer. To terminate the assay, cells were pelleted, followed by resuspension in ice-cold lysis buffer. Soluble cellular protein was subjected to G-LISA assay to detect GTP-Rac (A and B) and GTP-Cdc42 (C and D). Data from at least four separate experiments were integrated for the graphs shown. Error bars show SEM. NS, not significant; ^∗^p < 0.05, ^∗∗^p < 0.01.

Paks can be activated by Rac and Cdc42 (Bokoch, 2003). In the absence of convincing inhibitors for Rho family small GTPases, we determined the effect of PI3K inhibition with wortmannin upon Rho family activity in human neutrophils stimulated with iICs. The link between PI3K and Rac is well established, with several phosphatidyl-inositol-(3,4,5)-trisphosphate (PtdIns(3,4,5)P_3_)-activated Rac guanine nucleotide exchange factors (GEFs) described and shown to be functional in neutrophils (Dong et al., 2005, Kunisaki et al., 2006, Welch et al., 2002, Welch et al., 2005). Although iICs stimulated Rac in neutrophils, to our surprise, inhibition of PI3K did not reduce Rac activation in this context (Figure 5A). This contrasted the situation with fMLF-stimulated neutrophils, where Rac activation was dependent on PI3K (Figure 5B), in line with published observations (Welch et al., 2005). There is little evidence for PI3K-mediated regulation of Cdc42, and no PtdIns(3,4,5)P_3_-regulated Cdc42 GEFs have been described (Vermeren et al., 2009). Our experiments showed that Cdc42, like Rac, was activated in iIC-stimulated neutrophils and that Cdc42 activation was significantly reduced in neutrophils that had been pre-incubated with wortmannin (Figure 5C). Again, this contrasted our findings with fMLF-stimulated neutrophils, where, as expected, Cdc42 activation was PI3K independent (Figure 5D). We concluded from these experiments, that Cdc42 rather than Rac is regulated by PI3K in iIC-stimulated neutrophils and assumed that Cdc42, not Rac, is a likely regulator of Pak in iIC-stimulated human neutrophils.

### PI3Kβ/δ-Activated Erk Signaling Regulates Neutrophil Apoptosis

**Figure 6:**
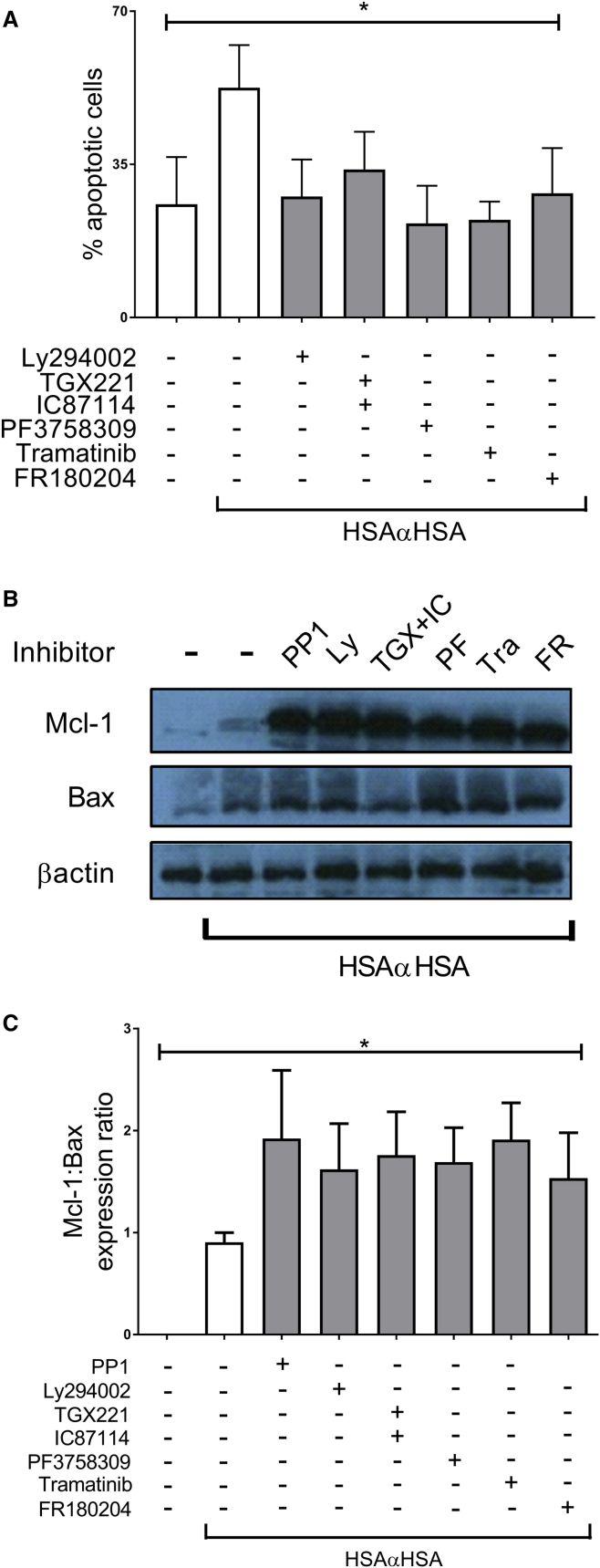
PI3K-Cdc42-Pak-Mek-Erk Signaling Regulates iIC-Induced Neutrophil Apoptosis Peripheral blood-derived healthy donor neutrophils were prepared and pre-incubated with small-molecule inhibitors (LY294002, stable pan-PI3K inhibitor; TGX221, PI3Kβ; IC87114, PI3Kδ; PF3758309, Pak; tramatinib, Mek; FR180204, Erk) or vehicle at 37°C for 10 min as indicated prior to stimulation with 10 μg/mL iICs or buffer. (A) Cells were cultured for 12 hr in Iscove’s Modified Dulbecco’s Medium (IMDM) supplemented with 10% autologous serum in a humidified, CO_2_ controlled incubator prior to staining with annexin V and propidium iodide for analysis by flow cytometry. Double-negative cells were defined as viable, annexin V-positive and propidium iodide-negative cells as apoptotic, and double-positive cells as necrotic. The same trends were observed with cells that had been cultured in PBS^2+^ instead of culture medium. (B) Following 3-hr culture in PBS^2+^, cells were pelleted, followed by resuspension in ice-cold lysis buffer. Soluble cellular protein was subjected to SDS-PAGE and western blotting to detect cellular Mcl-1 and Bax. A representative example is shown together with densitometry data integrated from a minimum of three separately conducted experiments. (C) The data shown are normalized to β-actin expression for ease of viewing. (A and C) The data shown are integrated from a minimum of three separately conducted experiments. Error bars show SEM. ^∗^p < 0.05, ^∗∗^p < 0.01. See also Figures S2 and S3.

iICs are known to trigger a number of functions in neutrophils, one of which is the induction of neutrophil apoptosis (Gamberale et al., 1998, Ottonello et al., 2001, Schettini et al., 2002). We therefore determined whether this unconventional signaling pathway regulates apoptosis. In line with published observations, we found that iIC-treated neutrophils exhibited accelerated apoptosis, with more than 50% of neutrophils undergoing apoptosis at 12 hr compared with approximately 25% of vehicle-treated neutrophils. Inclusion of inhibitors directed against PI3Ks, Pak, Mek, and Erk (FR180204) significantly reduced the extent to which iICs induced apoptosis (Figure 6A) and also secondary necrosis, as evaluated by flow cytometry and according to cytocentrifuge preparations (Figure S2). The inhibitors did not affect the extent of apoptosis in cells that had not been stimulated with iICs (Figure S2), demonstrating that this signaling pathway operates specifically following activation of neutrophils through their FcγRs.

Neutrophil apoptosis is known to be regulated by fine-tuning levels of Bcl-2 family proteins, where, in neutrophils, the pro-survival member Mcl-1 and the pro-apoptotic Bax are particularly important (Duffin et al., 2010, Murphy and Caraher, 2015). Stimulating neutrophils with iICs induced Bax expression, in agreement with an earlier report (Ottonello et al., 2001), and it also induced Mcl-1 (Figure 6B). Inhibitor treatment did not affect Bax levels but further increased Mcl-1 expression, thereby altering the ratio of Mcl-1 to Bax and delaying the induction of apoptosis (Figure 6C).

### PI3Kβ/δ Regulates Further Neutrophil Functions through Alternative Pathways

We next determined whether the unconventional signaling pathway also regulates other neutrophil functions, concentrating on ROS production, cytokine release, and L-selectin shedding.

**Figure 7:**
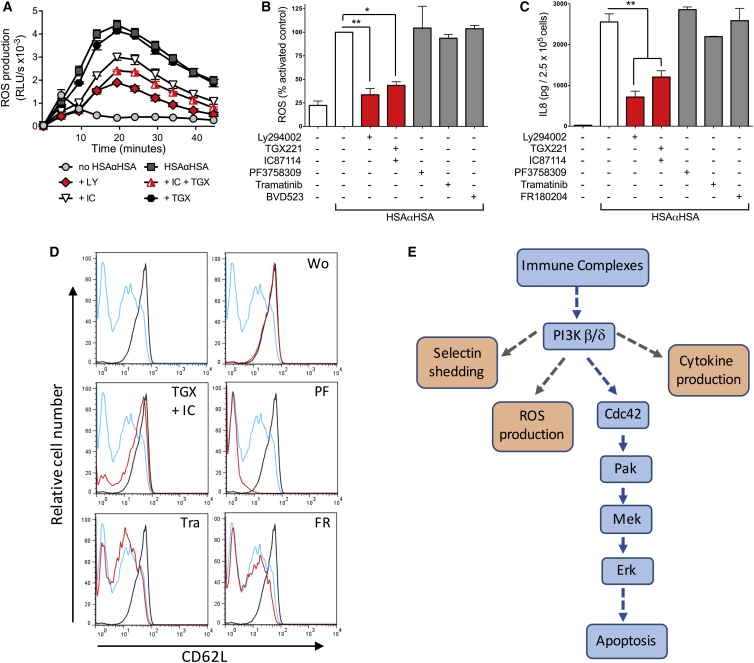
PI3Kβ/δ Signaling Regulates iIC-Induced Neutrophil Functions Other Than Apoptosis Peripheral blood-derived healthy donor neutrophils were pre-incubated with small-molecule inhibitors (LY294002, stable pan-PI3K inhibitor; TGX221, PI3Kβ; IC87114, PI3Kδ; PF3758309, Pak; tramatinib, Mek; FR180204 and BVD523, Erk) or vehicle at 37°C for 10 min as indicated prior to stimulation with 10 μg/mL iIC. (A and B) Characterization of iIC-induced internal ROS production. (A) The data (mean ± range) presented are from a representative experiment employing PI3K inhibitors of a total of three performed. (B) Total light emissions (mean ± SEM) of inhibitor-treated cells expressed as percentage of the response obtained with stimulated control cells. Data were pooled from a minimum of three separately conducted experiments. (C) Cells were cultured for 12 hr, followed by analysis of cytokine release in the culture supernatants by ELISA. The data shown were pooled from three separately conducted experiments. ^∗^p < 0.05, ^∗∗^p < 0.01. (D) Neutrophil surface CD62L was analyzed by flow cytometry. For ease of viewing, the mock-stimulated and iIC-stimulated histograms were copied into each inhibitor treatment (black, basal cells; blue, iIC-stimulated cells; red, iIC-stimulated and inhibitor-treated cells). A representative experiment is presented from a total of three separate experiments performed. (E) A schematic of the non-canonical signaling pathway regulating apoptosis in iIC-stimulated human neutrophils (blue boxes). This unusual pathway regulates Mek and Erk independently of Ras and Raf, instead using Cdc42 and Pak to regulate iIC-induced neutrophil apoptosis. Other functions are regulated by PI3Kβ/δ employing diverging pathways (orange boxes). See also Figure S4.

Stimulation of human neutrophils with iICs has been shown to stimulate the generation of intracellular ROS (Crockett-Torabi and Fantone, 1990, Fossati et al., 2002a), and iIC-induced ROS generation was shown to be required for the induction of apoptosis (Gamberale et al., 1998). In our hands, iICs drove significant production of internal ROS over an extended period of time (see Figure 7A for an example). Inhibition of PI3K using LY294002 attenuated iIC-induced ROS production. In keeping with the biochemical analysis shown in Figure 1, PI3Kδ was more critical than PI3Kβ for iIC-induced ROS production by human neutrophils (Figure 7A). To test whether PI3K regulates ROS production through the unusual pathway described in this work, we also pre-incubated neutrophils with Erk, Mek, and Pak inhibitors prior to analyzing ROS production. We used an alternative Erk inhibitor, BVD523 (Hayes et al., 2016) for this because the bright yellow color of FR180204 interfered with the assay (Figure S4). Inhibitors other than those for PI3K did not affect ROS production (Figure 7B).

Many physiological stimuli induce the production of inflammatory cytokines and chemokines by neutrophils to recruit immune cells to sites of inflammation and to regulate cross-talk between immune cells (Scapini et al., 2000). We noticed that stimulation of human neutrophils with iIC triggered strong IL-8 release (Figure 7C) but were unable to detect any tumor necrosis factor α (TNF-α) release (data not shown). iIC-induced IL-8 release was significantly reduced on pre-incubating the neutrophils with the pan-PI3K inhibitor LY294002 and when inhibiting PI3Kβ/δ but not Pak, Mek, or Erk (Figure 7C).

Finally we interrogated iIC-mediated L-selectin (CD62L) shedding, a sensitive indicator of neutrophil activation that is due to the rapid proteolytic cleavage of neutrophil L-selectin following a variety of stimuli. iICs induced efficient L-selectin shedding that was found to be dependent on PI3Kβ/δ but not on Pak, Mek, or Erk (Figure 7D). In conclusion, iICs drive multiple neutrophil functions that are regulated by PI3Kβ/δ-dependent pathways that diverge from the non-canonical pathways we have demonstrated to regulate apoptosis. These observations are in line with the notion that, on ligation of FcγR receptors, PI3K regulates diverse neutrophil functions by signaling through several different effector proteins (Figure 7E).

## Discussion

This work has unearthed an unorthodox signaling pathway that controls immune-complex-induced apoptosis in human neutrophils. Rather than employing the canonical and well characterized Ras-Raf-Mek-Erk module, our work demonstrates that an alternative PI3Kβ/δ-Cdc42-Pak-MekErk axis operates in iIC-stimulated human neutrophils (Figure 7). The present work was carried out under defined situations in vitro with neutrophils purified from the peripheral blood of healthy donors. It would be interesting to test whether the pathway that we have identified is also utilized under conditions that might be encountered by neutrophils at inflammatory sites in vivo. It is likely that the process of extravasation and the complex mixture of inflammatory cytokines and chemokines present at these sites prime and/or activate neutrophils, which may alter intracellular signaling pathways that are engaged.

PI3K regulates Erk activation in iIC-stimulated human and mouse neutrophils. As with human neutrophils, iIC stimulation drives PI3Kβ/δ-dependent apoptosis of mouse neutrophils (data not shown). We provide evidence that, although Pak acts as the MAP3K in human neutrophils, this is not the case in the mouse. Our data also indicate that signaling is not well conserved between fMLF-stimulated human and mouse neutrophils. Our findings are in line with a previous report that demonstrated differential usage of PI3K isoforms during ROS production by fMLF-stimulated human and mouse neutrophils (Condliffe et al., 2005). In the present work, we prepared human neutrophils from peripheral blood, whereas mouse neutrophils were derived from bone marrow preparations. Use of mouse bone marrow is common practice because it permits obtaining adequate numbers of cells for experiments. It is conceivable that the differences observed between human and murine neutrophils reflect a difference in the maturity of the cells. However, although human bone marrow-derived neutrophils have been shown to be functionally immature (Cowland and Borregaard, 1999), mouse bone marrow is reported to contain a large reservoir of functionally mature, readily primed neutrophils with regard to morphology and effector functions (Boxio et al., 2004, Itou et al., 2006). Our findings raise the possibility that there are genuine differences between signaling pathways used by mouse and human neutrophils. Primary human neutrophils can easily be prepared from peripheral blood but are not amenable to culture, transfection, or indeed transduction, restricting investigators to the use of inhibitors and functional assays. In allowing access to genetic manipulation and in vivo models, mice represent a very attractive alternative for neutrophil biology. The lack of conservation of signaling pathways between human and mouse coupled with the terminal differentiated and short-lived nature of neutrophils precludes any genetic analysis of the unconventional signaling that we describe for human cells. In addition, our observations, together with those of others, argue that novel insights gained with mouse models may need to be tested for their validity in human cells.

We identified Pak to act as an MAP3K in iIC-stimulated human neutrophils. Pak has been shown to act as an MAP3K in some circumstances, including in myeloid cells (Eblen et al., 2002, Smith et al., 2008). Interestingly, we observed Mek to be phosphorylated on Ser 217/Ser 221 in a Pak-dependent fashion (Figures 4A and 4D). These residues correspond to the Raf phosphorylation sites, with Pak reported to phosphorylate Mek on Ser 298 (Slack-Davis et al., 2003). Ser 293 phosphorylation has been proposed to sensitize Mek to Raf-dependent phosphorylation (Coles and Shaw, 2002, Frost et al., 1997). An alternative mechanism has also been proposed whereby phospho-Pak (Ser 293) is able to autophosphorylate Ser 217/Ser 221 (Park et al., 2007). In iIC-stimulated human neutrophils, Raf inhibition did not affect Erk activity. Instead, Pak inhibition interfered with Mek and Erk activation, in line with Pak-dependent Mek autophosphorylation (Park et al., 2007).

In contrast to the short-term inhibition of Erk activation by both Mek inhibitors (Figures 2M and 2P), at longer time points used for the examination of apoptosis, tramatinib but not AZD6244 was observed to be effective (Figure 6A; Figure S3). The Ras-Raf-Mek-Erk pathway is known to rely on a series of positive and negative feedback regulations that have been painstakingly deciphered in cancer cells (reviewed by Caunt et al., 2015). Tramatinib belongs to a new class of “feedback buster” Mek inhibitors that, in addition to inhibiting Mek, disrupt the conformation of the Mek activation loop, interfering with feedback-induced, Raf-mediated phosphorylation of Mek, thereby reducing feedback-induced Erk activation (Ishii et al., 2013). It is conceivable that Erk feedback loops operate in non-transformed neutrophils even under situations where the Ras-Raf-Mek pathway would not normally apply. Raf inhibition had no effect by itself, but it did when inhibition occurred in combination with AZD6244-mediated inhibition of Mek (Figure S3). One possibility is that, under the condition of long-term stimulation, a Raf-dependent input was precipitated because of triggering the negative feedback loop by Erk hypoactivity following Mek inhibition with the AZD6244 compound.

In a second, unexpected twist, our work advocates that Cdc42 rather than Rac is activated in a PI3K-dependent fashion in iIC-stimulated neutrophils. There have been rare examples of PI3K-dependent Cdc42 activation events (Aoki et al., 2004, El-Sibai et al., 2007), but no PIP3 activated GEFs have been identified yet. It will be fascinating to identify which GEF (and potentially additional intermediate steps) are involved in this reaction. It would also be interesting to identify whether PI3K-dependent Cdc42 activation is restricted to neutrophils.

Our work indicates that the unconventional PI3K signaling pathway delineated here is pro-apoptotic, complementing previous findings that described iIC-induced neutrophil apoptosis without elucidating the signaling pathway that regulates it (Gamberale et al., 1998, Ottonello et al., 2001, Schettini et al., 2002). Although iIC-induced apoptosis has been shown to depend on ROS production, our experiments demonstrate that apoptosis and ROS production are regulated by distinct pathways downstream of PI3K, suggesting that ROS production may be required but not sufficient for iIC-induced apoptosis to occur. Although it remains unclear whether there is a similar causal relationship between other immune-complex-induced functions that are regulated by PI3K (such as chemokine production) and apoptosis, the fact that distinct pathways regulate apoptosis and other effector functions may permit their pharmacological “uncoupling,” e.g., inhibiting apoptosis without affecting ROS production or chemokine release.

There is already evidence for pro- and anti-apoptotic functions of Erk (reviewed by Cagnol and Chambard, 2010). Our results suggest that PI3K/Erk-dependent regulation of Bcl-2 family members is required for iIC-induced pro-apoptotic effects. Erk is known to regulate Bcl2 family members, and our data agree with such a function in the context of iIC-induced neutrophil apoptosis.

Although better known for its anti-apoptotic function via Akt, PI3K has also been shown to have pro-apoptotic functions in TNF-α-stimulated neutrophils (Geering et al., 2011). It is interesting that neutropenia was one of the side effects reported with the PI3Kδ inhibitor idelalisib when administered in clinical trials to leukemia/lymphoma patients who had failed previous rounds of chemotherapy (Furman et al., 2014, Miller et al., 2015). In our experiments, inhibiting PI3Kδ did not drive apoptosis of control neutrophils (Figure S2J). Furthermore, the increased lifespan of neutrophils taken from chronic obstructive pulmonary disease (COPD) patients during acute exacerbations was also not affected by inhibition of PI3Ks (Juss et al., 2012). One possibility is that the neutropenia observed with idelalisib treatment may be due to effects on hematopoiesis and production or release of neutrophils into the circulation rather than on circulating neutrophils.

PI3K signaling holds a key role in controlling inflammation, including in neutrophil-driven chronic autoinflammatory diseases such as rheumatoid arthritis in which immune complexes represent an important pathophysiological mechanism. The recent development of genetically modified mice with altered PI3K signaling in combination with isoform-selective small-molecule inhibitors has led to a detailed understanding of PI3K signaling in a range of mouse models of autoimmune diseases. This has fed into the current focus on drug discovery surrounding PI3K to target human chronic autoimmune disorders in the clinical setting in addition to the important ongoing efforts in the cancer field. Our work highlights that PI3K signaling is not well conserved between mouse and human neutrophils, indicating that understanding derived from the mouse models needs to be interpreted with caution. In addition, given that the resolution of inflammation relies on the prompt induction of neutrophil apoptosis and efferocytosis of apoptotic neutrophils (Lucas et al., 2014, Poon et al., 2014, Rossi et al., 2006), our work has important implications for approaches to modulate inflammatory disease by targeting PI3Kβ/δ. Under conditions where immune complexes promote neutrophil apoptosis through the non-canonical pathway described here, inhibition of PI3Kβ/δ may act to limit neutrophil clearance. In contrast, under conditions where immune-complex-induced apoptosis is detrimental, for example by exceeding the clearance capacity and thereby leading to excess necrosis, inhibition of the pathway may be preferable.

## Experimental Procedures

### Reagents

Unless indicated otherwise, all reagents were obtained from Sigma-Aldrich. All reagents were of the highest available grade and lowest possible endotoxin level. Tissue culture media and buffers were obtained from Life Technologies, and dextran and Percoll were from GE Healthcare. The antibodies used were as follows: β-actin (rabbit polyclonal) from Abcam; Bax (clone B-9) and Mcl1 (rabbit polyclonal) from Santa Cruz Biotechnology; phospho-Akt Thr 308 (clone L32A4), phospho-Akt Ser 473 (clone D9E biotinylated), phospho-Erk Thr 202/Tyr 204 (clone E10), phospho-Mek1/2 Ser 217/221 (rabbit polyclonal), phospho-p38 Thr 180/Tyr 182 (rabbit polyclonal), and phospho-Pak1 Ser 144 (rabbit polyclonal) from Cell Signaling Technology; and CD62L-PC5 conjugate from Immunotech.

The inhibitors and final concentrations used were as follows: pan-PI3K, wortmannin (50 nM), and LY2940002 (10 μM, used for prolonged inhibition because wortmannin has a very short half-life in aqueous solutions); PI3Kα and A66 (10 μM); PI3Kβ and TGX221 (40 nM); PI3Kδ and IC87114 (1 μM); and PI3Kγ, AS252424 (30 μM), and CZC24832 (10 μM) (all from Sigma); the Raf inhibitor AZ628 (5 μM); the Mek inhibitors AZD6244 (1 μM) and trametinib (1 μM); the Pak inhibitors PF3758309 (5 μM) and IPA3 (10 μM); the Erk inhibitor FR180204 (10 μM); and BVD523 (5 μM) (all from Selleckchem).

### Human Peripheral Blood Neutrophil Isolation

Neutrophils were isolated from healthy donor blood as described previously (Lucas et al., 2013). Ethics approval was obtained from the local Lothian Research Ethics Committee (approvals 08/S1103/38 and AMREC 15-HV-013). Neutrophil purity was >95% as assessed by analysis of cytocentrifuge preparations.

### Isolation of Mouse Neutrophils

Mouse work was conducted under the control of the UK Home Office at the University of Edinburgh and approved by the University of Edinburgh animal welfare committee (PPL 60/4502). Mouse bone marrow-derived neutrophils were purified from C57Bl/6 mice using a discontinuous Percoll gradients as previously described (Gambardella et al., 2011). Neutrophil purity was 75%–80% as assessed by analysis of cytocentrifuge preparations. After washing, neutrophils were resuspended in Dulbecco’s PBS with Ca^2+^ and Mg^2+^, 1 g/L glucose, and 4 mM sodium bicarbonate (PBS^2+^).

### Preparation of Immune Complexes

iICs (human serum albumin [HSA] and rabbit polyconal IgG to HSA) were prepared in batches by titrating the point of equivalence between antigen and antibody and monitoring the point of equivalence by monitoring the absorbance at 450 nM as described previously (Crockett-Torabi and Fantone, 1990, Fossati et al., 2002a). Any soluble immune complexes formed were discarded prior to use in any experiment by repeated washing with PBS because insoluble and soluble immune complexes trigger distinct responses in neutrophils (Fossati et al., 2002a). The lowest concentration of iICs required to trigger a significant response was used for experiments (data not shown).

### Indirect Assaying of Protein Kinase Activity

Peripheral blood neutrophils were pre-warmed for 5 min at 37°C in PBS^2+^ before being incubated with inhibitors or vehicle. Pre-warmed stimuli were gently mixed with the pre-warmed neutrophils in a test tube. To end the assay, cells were pelleted and, after careful aspiration of the supernatant, lysed in ice-cold lysis buffer (20 mM Tris-HCl [pH 7.5], 150 mM NaCl, 1 mM EDTA, 1 mM EGTA, 1% Triton X-100, 2.5 mM Na pyrophosphate, 1 mM β-glycerophosphate, 1 mM Na orthovanadate, 0.1 mM PMSF, and 10 μg/mL each of antipain, aprotinin, pepstatin A, and leupeptin). After pelleting the detergent-insoluble material, lysates were boiled with sample buffer prior to resolution of proteins by SDS-PAGE, transfer to a polyvinylidene fluoride (PVDF) membrane (Millipore), and immunoblotting with antibodies of interest. Because of the strong proteolytic activities present in neutrophils, the numbers of experimental samples handled in any one experiment were kept to a minimum.

### Small GTPase Activity Assays

Ras, Rac, Cdc42, and Rho activities were determined by G-LISA (Cytoskeleton) essentially according to the manufacturer’s instruction, except that the lysis buffer was supplemented with 7 mM diisopropyl fluorophosphate in addition to the standard antiprotease cocktail.

### Assessment of Cell Viability and Apoptosis

Cellular changes associated with apoptosis of Diff-Quik-stained cytocentrifuge preparations were assessed by light microscopy. In addition, neutrophil apoptosis and secondary necrosis were measured by flow cytometry (FACS Calibur, BD Biosciences) of fluorescein isothiocyanate (FITC)-labeled annexin V-stained (Roche) and propidium iodide-stained (Sigma) cells as described previously (Lucas et al., 2013). Data were analyzed using Flowjo (Tree Star).

### Chemoluminescence Detection of ROS

Internal ROS production was measured by chemoluminescence in a Synergy H1 plate reader (BioTek Instruments) using luminescence-grade 96-well plates (Nunc) in PBS^2+^ supplemented with 150 μM luminol but in the absence of exogenous HRP. Following pre-incubation of neutrophils with inhibitors or vehicle (DMSO), stimuli were added manually, and light emission was recorded immediately. Data output was in relative light units (RLU) per second.

### Cytokine Release Assays

Following pre-incubation with inhibitors, neutrophils were stimulated as indicated and cultured in round-bottom 96-well plates (Corning Life Sciences) in RPMI 1640 medium supplemented with 10% autologous serum in a humidified tissue culture incubator at 37°C and 5% CO_2_. Supernatants were harvested for cytokine analysis by ELISA (R&D Systems) according to the manufacturer’s instructions.

### L-selectin Measurement

Surface L-selectin was determined by flow cytometry. Neutrophils were pre-incubated with inhibitors or vehicle, stimulated with iICs, and cultured at 37°C prior to incubation with FITC-conjugated antibody directed to L-selectin and analysis on a FACSCalibur flow cytometer (BD Biosciences). Data were analyzed using FlowJo software.

### Statistical Analysis

For pairwise comparisons, where data met the assumptions for parametric tests, two-tailed Student’s t tests were applied. Otherwise, the non-parametric Mann-Whitney rank-sum test was used for pairwise comparisons. For multiple comparisons, data were analyzed using Kruskal-Wallis one-way ANOVA on ranks with Dunn post hoc test. For kinetic experiments, the area under the graph was used for analysis. p values < 0.05 were considered statistically significant.

## Author Contributions

Conceptualization, S.V.; Methodology, J.Y.C., S.V., and I.D.; Investigation, J.Y.C. and S.V.; Writing – Original Draft, J.Y.C. and S.V.; Writing – Review and Editing, S.V., A.G.R., and I.D.; Funding Acquisition, S.V.; Resources, I.D. and A.G.R.; Supervision, S.V., A.G.R., and I.D.

## Supplemental Information

Document S1. Supplemental Experimental Procedures and Figures S1–S4

Document S2. Article plus Supplemental Information
