# Supplementary material for: Non-canonical PI3K-Cdc42-Pak-Mek-Erk Signaling Promotes Immune-Complex-Induced Apoptosis in Human Neutrophils
Source: Cell Rep. 2016 Oct 4;17(2):374–86. doi: 10.1016/j.celrep.2016.09.006 (PMC5067281; doi:10.1016/j.celrep.2016.09.006)
Supplement: Document S1. Supplemental Experimental Procedures and Figures S1–S4 [file mmc1.pdf]

Cell Reports, Volume *17*

Supplemental Information

Non-canonical PI3K-Cdc42-Pak-Mek-Erk Signaling Promotes Immune-Complex-Induced Apoptosis in Human Neutrophils

Julia Y. Chu, Ian Dransfield, Adriano G. Rossi, and Sonja Vermeren

Y. J. Chu et al, Supplemental Information.

Supplemental Figures and legends

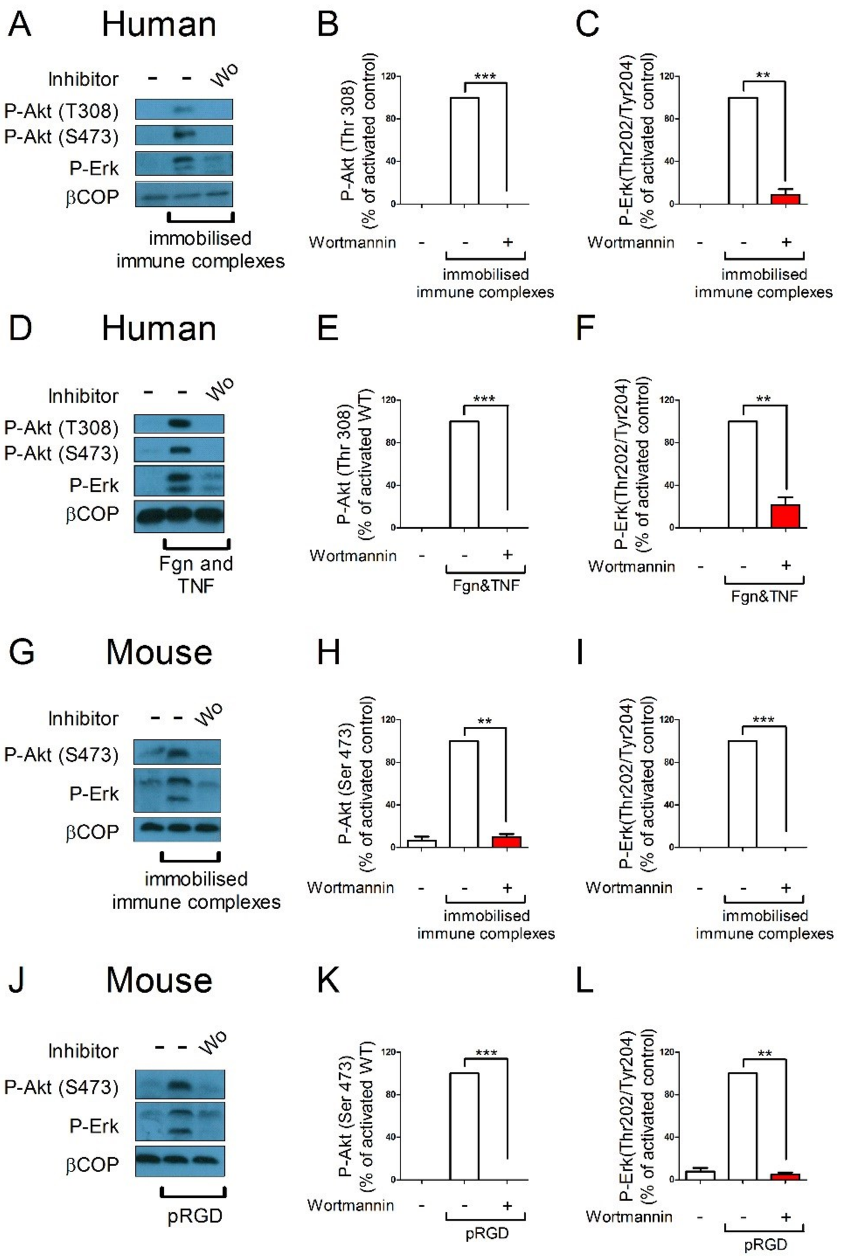

Figure S1 (related to Fig 1). Erk activation lies downstream of PI3K in human and mouse neutrophils on adhesion-dependent activation. Peripheral blood derived healthy donor neutrophils (A-F) and bone marrow-derived mouse neutrophils (G-L) were pre-incubated with the pan-PI3K inhibitor wortmannin or vehicle at 37ºC for 10 minutes as indicated prior to stimulation by plating onto BSA or immobilised immune complex-coated plastic; (A-C, G-I), fibrinogen (Fgn) in the presence or absence of TNF (D-F) or heat inactivated FBS or poly-Arg-Gly-Asp (pRGD; J-L) for 15 minutes. To terminate the assay, adherent and non-adherent cells were combined and scraped into ice-cold lysis buffer. Soluble cellular protein was subjected to SDS-PAGE and Western blotting to detect specific phosphorylation events or β-COP as a loading control as indicated. Representative examples (A,D,G,J) are shown together with densitometry data integrated from a minimum of 3 separately conducted experiments. For ease of viewing, data shown are normalized to the activated control and error bars show SEM; NS, not significant; **, p<0.01; ***p<0.001.

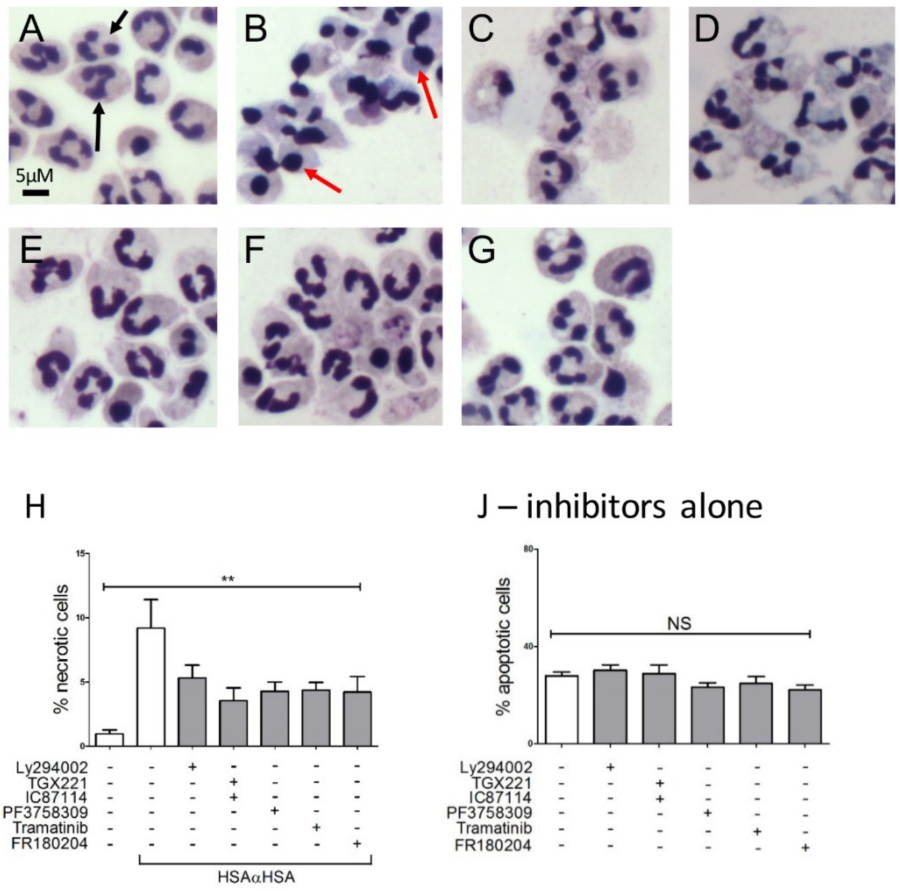

Figure S2 (related to Fig 6). PI3K-Cdc42-Pak-Mek-Erk signaling regulates iIC induced neutrophil secondary necrosis. Peripheral blood derived healthy donor neutrophils were prepared and pre-incubated with small molecule inhibitors or vehicle at 37ºC for 10 minutes prior to stimulation with 10 μg/ml iICs or buffer. Cells were cultured for 12 hours prior to cytocentrifuge preparation (A-G) or staining with annexin V and propidium iodide for analysis by flow cytometry (H,J). (A-G) Cytocentrifuge preparations. (A) cells only; (B-G) iIC stimulated cells; (B) vehicle; (C) LY294002; (D) TGX221 and IC87114; (E) PF76114; (F) Tramatinib; (G) FR180204. Scale bar, 5μm. (H, J) Flow cytometry. Viable cells were defined as double negative, apoptotic cells as annexin V-positive, propidium iodide-negative and necrotic cells as double positive. (H) Necrotic cells are plotted. (J) Cells were incubated with inhibitors, cultured and analysed by flow cytometry as detailed, but without stimulation with insoluble immune complexes. Data shown are integrated from a minimum of 3 separately conducted experiments. Error bars show SEM; **, $p<0.01$

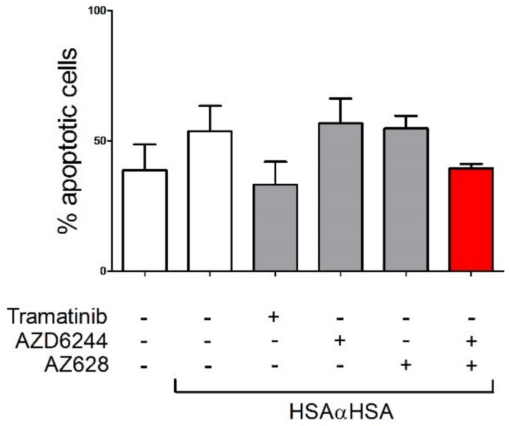

Figure S3 (related to Fig 6). Mek inhibition without 'feed-back buster' induces feed-back induced Raf activation during extended neutrophil incubation. Peripheral blood derived healthy donor neutrophils were prepared and pre-incubated with small molecule inhibitors or vehicle at 37ºC for 10 minutes as indicated prior to stimulation with 10 µg/ml iICs or buffer. Cells were cultured for 12 hours prior to staining with annexin V and propidium iodide for analysis by flow cytometry. Viable cells were defined as double negative, apoptotic cells as annexin V-positive, propidium iodide-negative and necrotic cells as double positive. Apoptotic cells are plotted. Data shown are integrated from a minimum of 3 separately conducted experiments. Error bars show SEM.

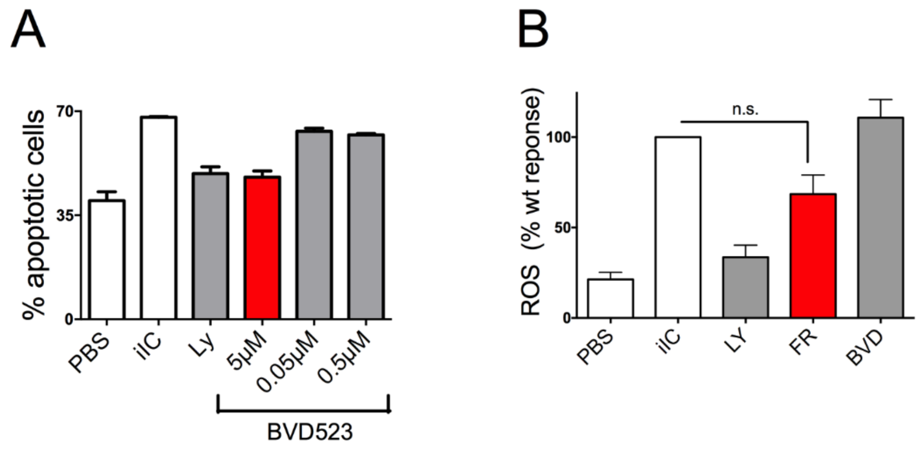

Figure S4 (related to Fig 7). Use of BVD523 as alternative Erk inhibitor for ROS assays.
The Erk inhibitor FR180204 is bright yellow, and caused an apparent inhibition in ROS assays. Apparent inhibitions had previously been observed with other brightly yellow coloured compounds (unpublished observation), prompting the search for an alternative Erk inhibitor. The colourless Erk inhibitor BVD523 is a candidate for use in the clinic, but its preclinical data are not very well established in the public domain (Germann et al., 2015; Hayes et al., 2016). Apoptosis experiments were carried out to establish its effective concentration. Peripheral blood derived healthy donor neutrophils were prepared and pre-incubated with small molecule inhibitors or vehicle at 37ºC for 10 minutes as indicated prior to stimulation with 10 µg/ml iICs or buffer. (A) Neutrophils were cultured for 12 hours prior to staining with annexin V and propidium iodide for analysis by flow cytometry. Viable cells were defined as double negative, apoptotic cells as annexin V-positive, propidium iodide-negative and necrotic cells as double positive. Apoptotic cells are plotted, identifying 5µM as the effective inhibitor concentration. Data shown are pooled from two separately conducted experiments. (B) Neutrophils were used in ROS production assays, where FR180204 apparently inhibited Erk, whilst 5µM BVD523 did not. The graph shown represents pooled data from three separate experiments; for ease of viewing data were normalized to the activated control; n.s., not significant.

Supplemental Experimental Procedures

Immobilized immune complex and fibrinogen coated dishes.

Preparation of plastic coated with immobilised IgG-BSA was done by coating 6cm tissue culture dishes with 100µg/ml fatty acid and endotoxin free BSA in PBS^{++} overnight at 4°C, followed by blocking with 1% fat free milk powder in PBS^{++} for 1 hour, extensive washing, incubation with rabbit anti-BSA antibody (1/2000; Sigma) for 1hour followed by further washes. BSA coated, blocked dishes were used as a negative control. For fibrinogen coated dishes, coating was overnight with 150µg/ml human fibrinogen (Sigma) in PBS^{++}. Where neutrophils were plated onto fibrinogen-coated plastic, they were co-stimulated with TNF for activation. Identically treated cells without co-stimulation served as negative control. Experiments were activated, harvested and lysed as described (Gambardella et al., 2011).

Supplemental References

Gambardella, L., Anderson, K.E., Nussbaum, C., Segonds-Pichon, A., Margarido, T., Norton, L., Ludwig, T., Sperandio, M., Hawkins, P.T., Stephens, L., *et al.* (2011). The GTPase-activating protein ARAP3 regulates chemotaxis and adhesion-dependent processes in neutrophils. Blood *118*, 1087-1098.

Germann, U., Furey, B., Roix, J., Markland, W., Hoover, R., Aronov, A., Hale, M., Chen, G., Martinez-Botella, R., Alargova, R., *et al.* (2015). The selective ERK inhibitor BVD-523 is active in models of MAPK pathway-dependent cancers, including those with intrinsic and acquired drug resistance. Cancer Research *75*, 4693.

Hayes, T.K., Neel, N.F., Hu, C., Gautam, P., Chenard, M., Long, B., Aziz, M., Kassner, M., Bryant, K.L., Pierobon, M., *et al.* (2016). Long-Term ERK Inhibition in KRAS-Mutant Pancreatic Cancer Is Associated with MYC Degradation and Senescence-like Growth Suppression. Cancer Cell *29*, 75-89.
